# The impact of novel coronavirus disease (COVID-19) on emergency and essential surgical care in Gedeo and Sidama zone hospitals: An institutional-based multicenter cross-sectional study

**DOI:** 10.1016/j.amsu.2022.103656

**Published:** 2022-04-22

**Authors:** Teshome Regasa, Abebayehu Zemedkun, Derartu Neme, Zemedu Aweke, Muddin Tadese, Hailemariam Getachew, Belete Alemu, Seyoum Hailu

**Affiliations:** a Anesthesia Department, Dilla University, Dilla, Ethiopia; b Anesthesia Department, Hawassa University, Hawassa, Ethiopia

**Keywords:** COVID-19 and emergency and important surgery, As well as the effect of COVID-19 on emergency and essential surgery, ACA Advanced Clinical Anesthesia, COVID Coronavirus disease, EESC Essential emergency surgical care, MSc Master of Science, SARS severe acute respiratory syndrome coronavirus, WHO World Health Organization

## Abstract

**Background:**

COVID-19 was initially detected in China's Wuhan, the capital of Hubei Province, in December 2019, and has since spread throughout the world, including Ethiopia. Long-term epidemics will overwhelm the capacity of hospitals and the health system as a whole, with dire consequences for the developing world's damaged health systems. Focusing on COVID-19-related activities while continuing to provide essential services such as emergency and essential surgical care is critical not only to maintaining public trust in the health system but also to reducing morbidity and mortality from other illnesses. The goal of this study was to see how COVID-19 affected essential and emergency surgical care in Gedeo and Sidama zone hospitals.

**Method:**

ology: A cross-sectional study was carried out in ten (10) hospitals in the Gedeo and Sidama zone. The information was gathered with the help of the world health organization (WHO) situational analysis tool for determining emergency and essential surgical care (EESC) capability. Infrastructure, human resources, interventions, and EESC equipment and supplies were used to assess the hospitals' capacity.

**Result:**

54.3% of the 35 fundamental therapies indicated in the instrument were available before COVID-19 at all sites, while **25.2 percent** were available after the COVID-19 pandemic. During the COVID-19 pandemic, there was a sharing of resources for treatment centers, such as a scarcity of oxygen and anesthesia machines, and emergency surgery was postponed. Before admission, the average distance traveled was 58 km.

**Conclusion:**

The COVID-19 pandemic, as well as existing disparities in infrastructure, human resources, service provision, and essential equipment and supplies, reveal significant gaps in hospitals' capacity to provide emergency and essential surgical services and effectively address the growing surgical burden of disease and injury in Gedeo and Sidama zone primary, general, and referral hospitals.

## Highlights

• 54.3% of basic interventions were available before COVID-19 at all facilities reduced to 25.2%.
• COVID-19 pandemic high effect on emergency and essential surgical cervices.
• Discrepancies in infrastructure, human resources, and other services also affect EESC.

## 1 Background information

Coronavirusoronavirus19 (COVID-19) was originally found in China in December 2019 in Wuhan, the capital of Hubei province, and has since spread worldwide. In December 2019, a new coronavirus (COVID-19) was discovered in three individuals with pneumonia who were linked to a Wuhan, China, cluster of acute respiratory disease cases. Several nations, including Europe, have sustained local transmission by the end of February 2020. Fever is the most prevalent clinical complaint in hospitalized patients, followed by cough, dyspnea, and myalgia, as well as weariness [1].

Coronaviruses are antisense RNA viruses of the Coronvirinae subfamily of the Coronaviridae family of the Nidovirales order. Only six distinct coronaviruses were known to infect people till December of 2019. In immune-competent adults, four of these viruses (HCoV-NL63, HCoV-229E, HCoV-OC43, and HKU1) normally cause mild common cold-like symptoms, but the other two have caused severe respiratory syndrome pandemics in the last two decades [2].

These viruses are ubiquitous in animals all over the world, but just a few cases have been reported in humans. COVID-19 was designated a pandemic by the World Health Organization (WHO) on March 11, citing approximately 118,000 instances of coronavirus disease in over 110 nations and territories around the world, as well as the ongoing risk of global spread [3].

Hospitals are an important aspect of the healthcare system because they provide necessary medical care to the public, especially in times of crisis. Long-term and simultaneous outbreaks can result in the progressive spread of disease, as well as quickly increasing service demands, which might overwhelm hospital and healthcare system capacity. During the current COVID-19 outbreak, disruption of these vital support services and supplies could jeopardize an unprepared healthcare facility's ability to provide acute health treatment. In addition, a significant proportion of employee absenteeism is likely. A scarcity of vital equipment and supplies could obstruct access to care and have a direct impact on healthcare delivery [4].

The term “essential and emergency surgical care” refers to surgical operations that are vital in averting premature death and disability in a specific ailment. Surgical treatments at referral hospitals are an important part of comprehensive health care. The worldwide burden of surgical disease is constantly rising, with low- and middle-income nations bearing a disproportionate share of the burden [5].

The fast-growing outbreak places an unprecedented strain on our healthcare system's effectiveness and long-term viability. The exponential increase in emergency department (ED) visits and inpatient admission volumes are two acute challenges [6].

Surgical disease has been estimated to constitute 11% of the total global burden of disease. But, Due to the pandemic of COVID-19, health facilities and the workforce are currently inundated by a plethora of activities related to controlling the pandemic. Essential health services, such as surgical services, that communities anticipate from the health system may be jeopardized as a result. Furthermore, health-seeking may be postponed due to social/physical distance requirements or community apprehensions about healthcare institutions being infected [7].

COVID-19 is expected to compromise emergency and essential surgical care, which means there is an increased risk of adverse outcomes by delaying surgical care for an undetermined period, due to a lack of focus, resource mobilization to COVID-19-related issues, fear of infection, availability of personal protective and infection prevention equipment, and so on. Focusing on COVID-19-related activities and continuing to provide essential services is critical not only to maintaining people's trust in the health system's ability to deliver essential health services, such as emergency and essential surgical care but also to reducing morbidity and mortality from other diseases [8].

## 2 Objectives of the study

### 2.1 General objective

To evaluate the impact of novel coronavirus disease-19 (COVID-19) on essential and emergency surgical care in Gedeo and Sidama zone hospitals from May 2020 to July 2020.

### 2.2 Specific objectives

To assess the impact of novel coronavirus disease-19 (COVID-19) on Emergency and essential surgical interventions.

To assess the impact of novel coronavirus disease-19 (COVID-19) on Emergency and essential surgical care equipment and supplies.

To assess the impact of novel coronavirus disease-19 (COVID-19) on the Availability of infrastructures.

To assess the impact of novel coronavirus disease-19 (COVID-19) on human resources.

## 3 Methodology

### 3.1 Study design and period

**Fig. 1:**
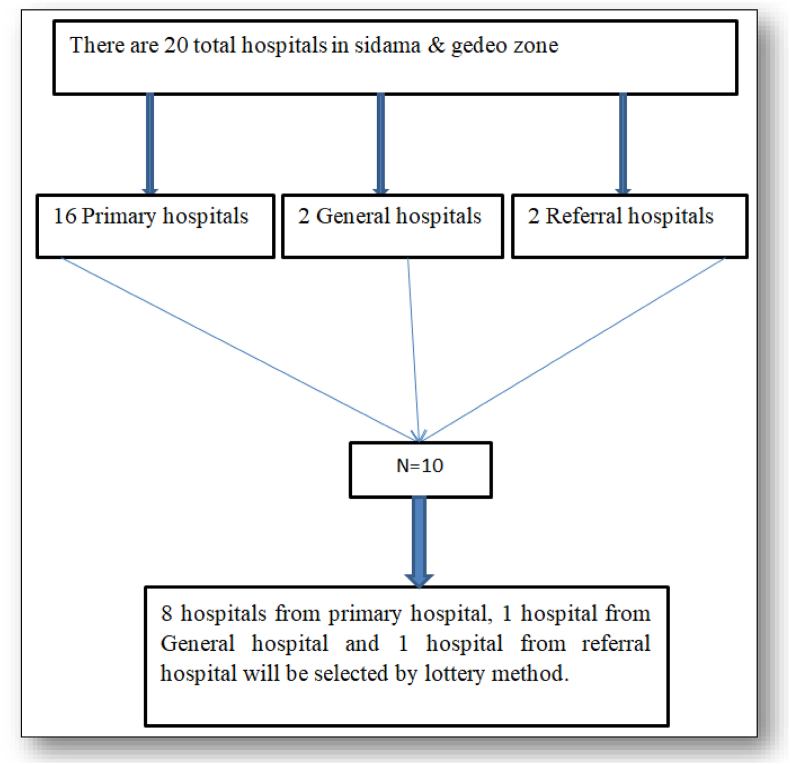
Sampling technique.

The institutional-based multi-center cross-sectional study was conducted from May 2020 to July 2020. Ethical clearance was taken from the Dilla University institution review board. Informed consent was taken from each hospital, which was included in the study unit. All hospitals in Gedeo and Sidama, which have active operating rooms, were included in the study, and Hospitals selected for the COVID-19 center were excluded from the study. Hospitals in the Gedeo and Sidama zone were categorized into clusters and ten hospitals were selected by lottery method as shown in Fig. 1 below. The WHO integrated management for emergency & essential surgical care (IMEESC) toolkit, Was used to assess emergency and essential surgical care (EESC) capacity among hospitals in Gedeo and Sidama zone hospitals. The capacity of the hospitals was evaluated before and after the COVID-19 by investigating four areas: infrastructure, human resources, interventions, and EESC equipment and supplies. The tool queries the availability of eight (8) types of care providers, 35 surgical interventions, and 67 items of equipment. The tool was distributed to ten public hospitals in Gedeo and Sidama zone and was completed in all health facilities by trained data collectors [9]. After completion of data collection, the data were manually checked for errors; and entered into SPSS version 20 for analysis. Descriptive statistics were used to determine indices both before and after COVID-19. . STROCSS 2021 checklist was used to state our completed activities by each item [10] and the manuscript registration number was 7676 https://www.researchregistry.com/browse-the-registry#home/.

## 4 Result and discussion

### 4.1 Result

Out of 10 hospitals, Dilla university referral hospitals had a high population service of around 750000 followed by adare general hospital with around 300000 population services as indicated in Table 1.

**Table 1:** Profile of surveyed hospitals. (n = 10).

Indicators name of the hospital	Name of hospitals									
	Dilla	Gedeb	Yirga chefe	Bule	Daye	Adare	hula	Chere	Chuko	Aletawendo
Estimated population served	750000	110000	160000	125000	130000	300000	170000	120000	150000	180000
Number of beds	165	35	55	45	40	155	65	47	65	70
Number of admissions (per year)	3500	450	620	500	550	2500	750	650	800	950
Number of functioning operating rooms	4	1	1	1	1	3	1	1	1	1
Number of patients requiring surgical procedures (per year)	900	90	120	85	95	750	130	75	135	145
Number of children (<15 years) requiring surgical procedures (per year)	120	30	45	40	35	110	55	30	48	54
Number of patients referred to a higher level of care for surgery (per year)	200	50	60	30	40	150	65	45	70	80
Average distance traveled to the facility (km)	300	35	25	23	18	150	28	22	30	45
Average distance traveled if referred elsewhere (km)	350	40	36	48	60	250	43	55	65	89

The majority of the general surgeons and obstetricians were from Dilla university referral and adare general hospitals, while clinical officers provided surgery at most of the primary hospitals in the Gedeo and Sidama zone. After COVID-19, there was a drop in the number of health workers since the susceptible groups needed relaxation, and some of them left their jobs out of dread of the pandemic as indicated in Table 2.

**Table 2:** Personal profiles in Gedeo and Sidama zone hospital.

Workforce	Total Health workers before COVID-19	Total Health workers after COVID-19
The general physician performing surgery	15	11
Anesthesiologists	1	0
Obstetrician	14	10
General doctors providing surgery	0	0
General doctors providing anesthesia	0	0
The clinical officer providing anesthesia	61	51
The clinical officer providing surgery	17	14
Midwives/paramedics	318	265

Regarding the availability of infrastructure & health resource during COVID-19 time, there were sharing of resource for the treatment center like the shortage of O2& anesthesia machine as indicated in Table 3.

**Table: 3:** Availability of infrastructures and health resources in Gedeo and Sidama zone Hospital s before COVID-19 and after COVID-19 (N = 10).

	Before COVID-19			After COVID-19		
	All the time	sometimes	Not available	All the time	sometimes	Not available
Oxygen cylinder	**10**		**0**	**7**	**3**	**0**
Running water	**7**	**3**		**6**	**3**	**1**
Electricity	**10**	**0**	**0**	**9**	**1**	**0**
Functioning anesthesia machine	**7**	**3**	**0**	**6**	**4**	**0**
Medical record	**10**	**0**	**0**	**10**	**0**	**0**
Blood bank	**10**	**0**	**0**	**10**	**0**	**0**
Hemoglobin and urine testing	**4**	**3**	**3**	**3**	**2**	**5**
Functional x-ray machine	**4**	**2**	**4**	**4**	**2**	**4**
Functional pulse oximeter	**10**	**0**	**0**	**8**	**2**	**0**
Management guidelines for anesthesia	**5**	**3**	**2**	**5**	**3**	**2**
Management guidelines for surgery	**6**	**3**	**1**	**6**	**3**	**1**
Management guidelines for emergency care	**5**	**0**	**5**	**5**	**0**	**5**
Management guidelines for pain relief	**8**	**0**	**2**	**8**	**0**	**2**
The area designated for emergency care	**10**	**0**	**0**	**10**	**0**	**10**
The area designated for post-operative care	**6**	**0**	**4**	**4**	**0**	**6**

**Table: 4:** Availability of surgical interventions in Gedeo and Sidama zone Hospital before COVID-19 and after COVID-19 (N = 10).

	Before COVID-19		After COVID-19	
	available	Not available	available	Not available
Resuscitation (airway, hemorrhage, peripheral percutaneous intravenous access, peripheral venous cut down)	10	0	10	0
Cricothyroidotomy/Tracheostomy	4	6	2	8
Chest tube insertion	5	5	3	7
Removal of foreign body (throat/eye/ear/nose)	10	0	4	6
Acute burn management	10	0	10	0
Incision and drainage of abscess	10	0	10	0
Suturing (for wound, episiotomy, cervical and vaginal laceration)	10	0	10	0
Wound debridement	10	0	10	0
Cesarean section	10	0	7	3
Dilation and curettage/vacuum extraction	10	0	10	0
Obstetric fistula repair	2	8	1	9
Tubal ligation/vasectomy	6	4	2	8
Biopsy(lymph node, mass, other	4	6	2	8
Appendectomy	10	0	5	5
Hernia repair	10	0	1	9
Hydrocelectomy	10	0	7	3
Cystostomy	10	0	5	5
Urethral stricture dilation	2	8	0	10
Laparotomy	10	0	7	3
Male circumcision	10	0	0	10
Neonatal surgery(abdominal wall defect, colostomy, imperforate anus, intussusception	2	8	2	8
Cleft lip repair	1	9	0	10
Club foot repair	3	7	0	10
Contracture release/skin graft	4	6	1	9
Closed treatment of fracture	10	0	10	0
Open treatment of fracture	10	0	5	5
Joint dislocation treatment	10	0	10	0
Drainage of osteomyelitis	10	0	4	6
Amputation	3	7	2	8
Cataract surgery	1	9	0	10
Regional anesthesia block	3	7	3	7
Spinal anesthesia	10	0	10	0

Before COVID-19, **54.3 percent** of all facilities offered basic surgical interventions; after COVID-19, **25.2 percent** of all facilities offered basic surgical operations. Due to staff leave and some equipment sharing for a treatment center, there was a significant variance in surgical intervention following COVID-19. Emergency surgery, such as appendicitis, emergency C/S, and foreign body removal, is commonly referred to referral hospitals as indicated in t able4.

There was frequently a shortage of capital outlays for resuscitation secondary to lock down & sharing equipment during the COVID-19 pandemic in Gedeo and Sidama zone hospitals (see Table 4). Face masks, airway & disposable gloves were frequently in shortage as indicated in Table 5.

**Table 5:** Availability of capital outlays for resuscitation in Gedeo and Sidama zone Hospital before COVID-19 and after COVID-19 (N = 10).

	Before COVID-19			After COVID-19		
	absent	Available with a frequent shortage	Fully available	absent	Available with a frequent shortage	Fully available
Resuscitator bag valves & mask(adult)	0	1	9	0	4	6
Resuscitator bag valves & mask (pediatrics)	0	2	8	0	5	5
Stethoscope	0	0	10	0	0	10
Suction pump with a catheter		2	8	0	4	6
Blood pressure measuring equipment	0	0	10	0	0	10
Thermometer	0	3	7	0	3	7
Scalpel with blades	0	2	8	0	2	8
Retractor	0	4	6	0	4	6
Scissor	0	4	6	0	4	6
Oropharyngeal airway (adult size)	0	3	7	0	8	2
Oropharyngeal airway (pediatric size	0	4	6	0	7	3
Forceps, artery	0	5	5	0	5	5
Glove (sterile)	0	3	7	0	7	3
Glove (examination)	0	4	6	0	8	2
Needle holder	0	3	7	0	3	7
Sterilizer	0	7	3	0	7	3
Vaginal speculum	0	5	5	0	5	5

**Table 6:** Availability of renewable supplies for resuscitation in Gedeo and Sidama zone Hospital before COVID-19 and after COVOD-19 (N = 10).

	Before COVID-19			After COVID-19		
	Absent	Available with frequent shortage	Fully available	Absent	Available with frequent shortage	Fully available
Nasogastric tubes	0	0	10	0	0	10
Light source	0	1	9	0	1	9
The intravenous fluid infusion set	0	0	10	0	0	10
Intravenous cannula	0	0	10	0	2	8
Syringes with a needle(disposable)	0	0	10	0	3	7
Sharps disposal container	0	0	10	0	2	8
Tourniquet	0	3	7	0	3	7
Needles and sutures	0	0	10	0	6	4
Splints for arm, leg	2	5	3	2	5	3
Urinary catheter	0	0	10	0	0	10
Waste disposal container	0	3	7	0	5	5
Face mask	0	2	8	0	4	6
Eye protection	4	2	4	4	2	4
Protective gown/aprons	1	3	6	2	8	0
Soap	0	2	8	0	4	6

Endotracheal tubes & laryngoscopy were frequently in shortage in 6 & 8 hospitals respectively after COVID-19 (see Table 6). There was no fully available infuser bag in all hospitals as indicated in Table 7.

**Table: 7:** Availability of supplementary equipment for resuscitation in Gedeo and Sidama zone Hospital before COVID-19 and after COVOD-19 2020 (N = 10).

	Before COVID-19			After COVID-19		
	Absent	Available with a frequent shortage	Fully available	Absent	Available with frequent shortage	Fully available
Magill forceps(adult)	5	2	3	5	2	3
Magill forceps(pediatrics)	6	2	2	6	2	2
Endotracheal tubes(adult)	0	3	7	0	6	4
Endotracheal tubes(pediatric)	0	4	6	0	6	4
IV infuser bag	5	3	2	5	5	0
Chest tubes insertion equip	1	3	6	3	6	1
Laryngoscope (adult)	0	5	5	0	8	2
Laryngoscope (pediatric)	0	3	7	0	5	5
Cricothyroidotomy set	6	2	2	6	4	0

### 4.2 Discussion

Our findings demonstrate that before COVID-19, only **54.3 percent** of fundamental surgical interventions were available at all facilities; after COVID-19, only **25.2 percent** of basic surgical interventions were available at all facilities. Due to staff departure and some equipment sharing for a treatment center, there was a large variance in surgical intervention following COVID-19. Emergency surgery, such as appendicitis, emergency C/S, and foreign body removal, is frequently referred to referral hospitals. Our findings demonstrate that sharing resources for a treatment center, granting COVID-19 breaks to vulnerable employees, and resource discrepancies harmed emergency and vital surgical services in the southern part of Ethiopia. We were getting the report from 8 primary hospitals 15 emergency patients died on the road during traveling to referral hospitals secondary to hemorrhage and septic according to the report. This finding is consistent with the findings of a scoping review study conducted in India by K.Soreide et al. on the long and short-term impact of COVID-19 on surgical care [11].

Correctional survey studies show that Five million individuals around the world were estimated to be without timely, safe, and cheap surgical and anesthetic services. This situation is exacerbated in low and middle-income countries, where 6.5% of all procedures are conducted in the poorest countries. In low-income countries, improving surgical access requires a systems-based approach that addresses infrastructural deficiencies, trained/skilled staff, suitable equipment and drugs, and necessary and emergency surgical treatment [12].

Complete disregard for emergency and vital surgical services would be regarded as undesirable collateral damage, increasing the number of lives and life-years lost as a result of the COVID-19 pandemic accidently. When surgical theatres are shut down or reduced to a minimum of activity, and triage for the recommended and urgent surgeries is necessary, this might present ethical difficulties in a time of limited resources and heavy strain on critical care workers [13].

This study found critical shortcomings in basic infrastructure and competent human power required to offer surgical services in line with a crossectional survey study in Sub-Saharan Africa, which has been well reported in earlier studies. The equipment was mostly insufficient, with considerable gaps in the availability of running water, hemoglobin and urine testing, a working x-ray machine, and a working pulse oximeter. Essential equipment was not always present at all of the sites, according to the report. During the epidemic, systemic changes that address human resources, supplies/equipment, and infrastructure, as well as other existent problems in the resource-limited area, were exacerbated [14].

A single-center observational cohort study by McLean, R.C., et al. state that The number of patients requiring surgery varied greatly between facilities, which might be explained by differences in the presence of qualified surgeons and obstetricians, as well as the burden of trauma cases. In these studies, surgical procedures were performed by non-physician clinical officers instead of qualified doctors even before the COVID-19 pandemic. Before COVID-19, most primary hospitals did not offer obstetric fistula repair, urethral stricture dilation, cleft lip repair, clubfoot repair, or cataract surgery [15,16].

A retrospective comparative study by Ciarleglio, F.A., et al., shows that Lockdown procedures made it impossible for some patients to get to the emergency room, and the worry of contracting COVID-19 in the hospital added to the difficulty. Furthermore, the socioeconomic ramifications of public health interventions are likely to contribute to poor long-term health outcomes, and the COVID-19's effects reduced the quality of surgical care, with poorer prognosis and greater morbidity rates. Due to delayed emergency department access and a “filter effect” caused by public fear of COVID-19 infection, only the most serious cases reached the emergency department on time, which was in line with our study [15,16].

## 5 Implications of study

This finding indicates that primary hospitals previously lacked EES services, and the inclusion of the COVID-19 pandemic further harmed the service, implying that the ministry of health should establish guidelines for dealing with similar pandemics. Of course, the ministry of health developed unique surgical system development methodologies that focus on saving lives through safe surgery (SaLTS). At all levels of the healthcare system, this national initiative aims to improve access to safe, required, and emergency surgical and anesthetic services. So far, no comprehensive assessment of surgical care capability in Ethiopia has been conducted in terms of physical resources and service supply at various levels of the health care system, especially in primary hospitals which were found in rural areas [17]. Therefore, this finding gives a clue for the development of new guidelines or improving this SaLTS system for the early management of such conditions.

## 6 Challenge

The main challenge during this project was a lack of COVID-19 personal protective equipment, and during data collection, the hospital management and several assistant managers tried to provide us with false information to hide their weaknesses. We solved the scarcity of personal protective equipment by communicating with various organizations, finally, Dilla university helped us by supplying important equipment, and we solved the data collection challenge by asking professionals who work in specific departments, visiting infrastructure blindly, and maintaining smooth communication with staff who are heavily involved in the management of the EES service area.

## 7 Conclusion

The COVID-19 pandemic, as well as existing disparities in infrastructure, human resources, service provision, and essential equipment and supplies, show that hospitals in the Gedeo and Sidama zone have significant gaps in their ability to deliver EESC and effectively address the growing surgical burden of disease and injury. The necessity for continuing investment in EESC infrastructure, equipment, and supplies, as well as adequately qualified surgeons, anesthesiologists, and obstetricians/gynecologists, is highlighted in this study.

## Ethical approval

Ethical approval was taken from Dilla University institutional review board.

## Sources of funding

10.13039/501100021788Dilla university.

## Author contribution

Teshome Regasa and Abebayehu Zemedkun, and Derartu Neme, contributed to study conception, collected data, prepared manuscript, and performed statistical analysis. Zemedu Awoke, Belete Alemu, Muddin Tadese, Seyoum Hailu, and Hailemariam Getachew contributed in statistical analysis. All the authors read the manuscript and approved the final submission.

## Trial registry number

The research were registered on research registry platform with a unique identification number of researchregistry7676.

## Guarantor

Teshome Regasa.

## Consent

Informed consent was taken from each included hospital.

Dilla University funded us 200 dollars for the research project. The sponsor has no role in the research activity. The sponsor did not take part in any action of the research project other than funding.

## Provenance and peer review

Not commissioned, externally peer-reviewed.

## Declaration of competing interest

There is no conflict of interest to declare.
